# ARTYCUL: A Privacy-Preserving ML-Driven Framework to Determine the Popularity of a Cultural Exhibit on Display

**DOI:** 10.3390/s21041527

**Published:** 2021-02-22

**Authors:** Gatha Tanwar, Ritu Chauhan, Eiad Yafi

**Affiliations:** 1 Amity Institute of Information Technology, Amity University, Noida 201313, India; gatha.tanwar@gmail.com; 2 Center for Computational Biology and Bioinformatics, Amity University, Noida 201313, India; 3 Malaysian Institute of Information Technology, Universiti Kuala Lumpur, Kuala Lumpur 50250, Malaysia

**Keywords:** internet of things (IoT), cultural heritage, artifact reputation, histogram of oriented gradient (HOG), clustering, human detection, human density

## Abstract

We present ARTYCUL (ARTifact popularitY for CULtural heritage), a machine learning(ML)-based framework that graphically represents the footfall around an artifact on display at a museum or a heritage site. The driving factor of this framework was the fact that the presence of security cameras has become universal, including at sites of cultural heritage. ARTYCUL used the video streams of closed-circuit televisions (CCTV) cameras installed in such premises to detect human figures, and their coordinates with respect to the camera frames were used to visualize the density of visitors around the specific display items. Such a framework that can display the popularity of artifacts would aid the curators towards a more optimal organization. Moreover, it could also help to gauge if a certain display item were neglected due to incorrect placement. While items of similar interest can be placed in vicinity of each other, an online recommendation system may also use the reputation of an artifact to catch the eye of the visitors. Artificial intelligence-based solutions are well suited for analysis of internet of things (IoT) traffic due to the inherent veracity and volatile nature of the transmissions. The work done for the development of ARTYCUL provided a deeper insight into the avenues for applications of IoT technology to the cultural heritage domain, and suitability of ML to process real-time data at a fast pace. While we also observed common issues that hinder the utilization of IoT in the cultural domain, the proposed framework was designed keeping in mind the same obstacles and a preference for backward compatibility.

## 1. Introduction

As any civilization progresses, it also tries to preserve and promote the heritage of its older generations. Be it art, music, or monuments, these together forms the cultural heritage in which nations take pride. The museums and heritage sites not only form the backbone of cultural heritage (CH), but also contribute to the country’s tourism industry. With advancements in technology, there is a world of possibilities for its application for CH promotion among the masses, and to introduce it to the new generation. The CH domain therefore offers a wide scope for applications of technology to further its cause [1,2,3].

An IoT-based smart ecosystem is made up of a multitude of sensors, audio-visual interfaces and a local network based on protocols such as Bluetooth, ZigBee, 6LoWPAN, Radio-Frequency Identification (RFID), 3G/4G GSM, and others [4,5]. The sensors are used to continuously monitor the physical environment and trigger events based on their measurements. Such events are communicated on the local network to other sensors or cause human-discernible actions on the audio-visual interfaces. When we talk about a CH site, these sensors offer a powerful means to control and monitor the ambience of the premises. While preservation of heritage items is paramount and often plagued by shortage and inexperience of the staff, a sophisticated artificial intelligence (AI)-driven smart network can be a powerful tool to detect and predict events of interest [6,7].

Apart from monitoring and control of ambience factors, internet of things (IoT) technology has been utilized along with social networking and artificial intelligence to personalize a visit experience. A noteworthy example is Museon in Den Haag, the Netherlands [8]. This museum had utilized a Raspberry Pi-based interaction and logging system to provide visitors with an avenue to share their post-visit experience. It had been observed that the presence of interactive add-ons not only stimulated the visitors but also facilitated networking between exhibits and across sites. The visitors were able record and share experiences over their social networks; therefore, the heritage sites were linked with similar exhibitions at other locations. Such studies not only helped with fine-tuning the visit personalization procedures, but also helped museums to tailor guided tours and events to attract more visitors.

A majority of visitors have a specific interest in mind and may be open to recommendations, or geographic directions. IoT-based technology can be employed to develop smart systems to gauge user interest and display relevant information related to the exhibit [9,10,11]. One of the major factors that drive the modeling of a recommender system is the awareness of the reputation of the exhibits. Such recommender systems can guide visitors to related items within or nearby the premises. For example, if the paintings by artist Vincent Van Gogh were attracting more visitors at a museum, then an in-house recommender system can display the location and information of other local museums that were displaying works by the prolific artist. This will facilitate a better espousal of CH; small-scale museums could be endorsed by the more popular destinations.

Another aspect of application of IoT to CH sites is the analytics of the data gathered by the installed devices [12,13]. The various IoT devices installed at such a site may include security cameras, temperature and ambience sensors, and logging systems for visitors and so on. Such devices generate a large volume of data that may remain untapped. The analytics of this data can uncover visit patterns [14], popularity of artifacts, and other information. The proposed framework, ARTifact popularitY for CULtural heritage(ARTYCUL) is an endeavor in this area of interest. We have investigated the analytics offered by the streams of videos captured by security cameras that watch over the exhibits. For this, a model was designed and implemented that aggregated densities of the visitors through detected human figures in the video and thus estimated the popularity of exhibits.

This paper covers investigation into the current trends in usages of IoT in cultural heritage domain. We also delve into how IoT has found applications so as to provide a better visiting experience. We also discuss the major challenges faced by these applications and how we have tried to overcome the same. Further sections delve into the determination of the popularity of artifacts and display exhibits in a premise. The reputation of any display exhibit is a function of many factors, be it the visiting behavior of the masses, the placement of the particular artifact, or the particular category or culture to which it belongs. ARTYCUL was designed to generate a graphical representation of visitor trends and densities using security cameras streams. Finally, we discuss the approach with respect to the limiting cases and the advantages ARTYCUL would offer to the management team of museums.

## 2. Related Work

### 2.1. The Existing Applications of IoT in Cultural Heritage Domain

IoT has found a multitude of applications in the field of preservation and promotion of cultural heritage. Research is being conducted to maximize the impact of this technology. A notable area of research is the development of smart spaces within museums [14]. These smart spaces are made up an ecosystem formed by ambience sensors, RFID devices, Bluetooth devices, and various other technologies to detect and communicate with devices owned by the visitors, or the interfaces installed in the premises. In this context, IoT acts as a bridge between the vast knowledge base and its tangible representation to the user. This not only helps in fostering the knowledge, but also leads to a more enjoyable experience.

The sensor nodes which are a major component of such smart systems are being further enhanced in concurrence with the developments in integrated circuit technology and faster networking protocols. These nodes are being designed to support a more flexible configuration and ability to adapt to the changing needs of the systems. Such sophisticated sensor nodes facilitate a refined level of monitoring of the physical environment, creation of faster and multiple connections, and the handling of larger volumes of data. One of the successful examples is the user experience being presented at Maschio Angioino castle in Naples [7]. The premises at this cultural site are a well-connected network, capable of quick communication at various ends. Another case of interest is the highly interactive display at Exploratorium in San Francisco [15]. The smart nodes comprising a smart network at a heritage site also need to be in step with the rich content which is being distributed through the network. Museum curators and designers who are more aware of the expected outcome from such systems could therefore be taught how to configure and leverage such smart objects to their designs. Research around the definitions of the semantics of smart objects will allow them to be used beyond their potential and help better adoption in the cultural heritage domain. An important factor of the smart experiences should be that they do not overwhelm the non-technical users and thus diminish the intended impact.

Museums and heritage sites also need to gauge the interests of the visitors so as to promote themselves and to tap the population with similar interests. When a visitor comes to a heritage site, there are two possibilities: they are interested in some particular civilization, or time period, or artist; or they are present to explore what is on display and do not have any specific preferences. IoT-based systems can be used to interact with the visitors and guide them around the premises [16]. At a personal level, a user will be more comfortable with a museum guide which can provide unbiased and correctly sourced historical background, can be turned off when required. The system can be further refined to provide information about the specific exhibits as well as the whole premises. When a user is interacting with such a system, they are implicitly providing insight into their visiting behavior. The museum curators and planning staff can utilize this information to better organize the premises. The knowledge of such patterns may also help in planning the visit schedules and guided tours. Such systems have been achieved using location aware services [17] and handheld devices [18,19] at many museums. Research focused on the adoption of location aware services have defined a framework which primarily consists of an ecosystem of wireless networks, for instance a Bluetooth Low Energy (BLE) or RFID infrastructure, which would mark the bounds for tracking, a device or service at the end of the visitor to publish their location, and finally, software which would generate actions on receiving the updated location messages. The actions could include some response back to the device carried by the visitor, or the location could be forwarded to other components of the smart system. In such a setup, the movements of a user are tracked, and the system generates events to enhance the experience by providing further details. The vast reach of social networking may be used in conjunction to reach out to a visitor’s social circle. It should be noted that this kind of system will access a visitor’s personal profile. The information which has been collected about the visitor can be used by augmented reality (AR) applications to draw up richer and more personalized content, but such a system would be required to address user privacy concerns and maintenance requirements of the sophisticated hardware.

Research has also delved into the possibilities of analytics of vast amount of data generated by an IoT-based smart environment. There have been attempts to utilize the deployed environment to compute a reliable interpretation of the relationship between the visitor behavior and the displayed exhibits [20]. Systems have been tailored to classify visitor behavior and use this knowledge to determine the popularity of the cultural artifacts on display. Therefore, such systems not only support the enhancement of the cultural experiences, but also of the level of interest that the artifacts command. In Table 1, the application in retrospectives of cultural heritage is discussed widely. Such systems are the basis of real-time recommender systems [10,11,21], which consider the explicit and implicit preferences of a visitor and direct them to exhibits of similar interests. Some recommender systems are also sophisticated enough to draw up a tour map for the visitor’s area of interest. Moreover, the organization and layout of a museum or visit tours can be optimized to suit the resulting trends. The proposed system aims at computing and utilizing similar analytics of the visiting patterns.

### 2.2. Constraints in IoT Utilization

Some of the major concerns for the setup of IoT systems at a heritage site arise from the fact that such places might be remotely located and mostly not connected with high-speed internet [8]. A museum might invest in a good internet connection and build a private network to connect the devices in the premises. This is also more helpful to push information brochures and guidance mobile applications on the visitor’s mobile phones. However, when we consider a heritage site or monuments, these are mostly far from cities and have a limited connectivity.

Another constraint that comes to the fore is visitor privacy concerns. Some of the smart systems at a heritage site aim at providing a personalized experience. This requires the visitor to share their details before or after the visit. In some cases, the smart system may connect with a visitor’s mobile phone for a better coordination of the visit [21,22]. This may result in the service accessing the user’s social profiles. Some visitors might be wary of sharing their details and might prefer a more anonymous approach to a better service. While the mobile phone owners can take the precautions of setting access permissions to the mobile phone system, they also need to be wary of the policies and terms of conditions during installation. The mobile application can be uninstalled when the user exits the premises. Moreover, a majority of visitors are one-time visitors and the quality of information they provide may not be correct or complete.

The proposed framework is a lightweight approach that addresses the above concerns. The system would work well over a low-speed internet. The visitors will not be tracked, and only the visitor density around the display exhibits will be tracked by the camera component of the framework. The principle underlying functions of ARTYCUL is backward compatibility; it can be used with CCTV streams provided by the existing security system. It is easy to install and the maintenance which it may require is that of software updates and the existing security cameras, thereby addressing the installation and maintenance concerns.

### 2.3. Motivation and Contribution

The advent of IoT technology in the cultural heritage (CH) domain is an exciting avenue for researchers. It not only pushes researchers to explore new applications of the technology, but also further the cause of heritage promotion among the masses. The study of existing solutions led the authors to the realization that a backward-compatible monitoring solution capable of providing privacy-assurance to the site visitors is lacking in the CH domain. The gap area was apparent because most of the IoT systems deployed on sites require user profiling, or the installation of sophisticated smart devices. To the best of our knowledge, ARTYCUL is a novel ML-driven framework that does not profile users or require their personal data. Moreover, it needs not to engage the visitor to determine the popularity garnered by a display item. The framework uses video streams of CCTV cameras, meaning there is no need for the installation of any other hardware. Therefore, this is a plug-and-play solution for curators of museum or heritage sites to determine how well the artifacts have been placed at a site under their supervision.

## 3. Materials and Methods

### 3.1. Determination of Artifact Popularity

#### 3.1.1. Visitor Behavior Classification

Museums and heritage sites aim at attracting a large number of visitors and to provide them a fulfilling visit experience. Understanding the visiting styles of prospective visitors helps in a better organization of the artifacts and the planning of guided tours. A relevant tour of the premises ensures that the visitor leaves with a positive feeling and relays good feedback to others.

Research work has tried to classify the visiting behavior on the basis of three parameters. Firstly, the percentage of the artworks that have been viewed. Secondly, the average time the viewer has spent on the viewed artwork; this is the time a viewer has spent in interacting with the exhibit directly, as well as with an accompanying multimedia interface. This parameter can be considered a part of the purpose of the visit. Thirdly, the order of the path covered by the viewer. Using these three factors, visitor behavior can be divided into four major categories [23,24]. These are classified by animals, namely, an ant, a butterfly, a fish and a grasshopper. A visitor with the visiting behavior of an ant follows the planned path around the exhibits, thus viewing a large number of items, and has a high probability of using the recommender systems. A visitor characterized as a butterfly tends to be guided by the placement and orientation of the display exhibits, and would stop to spend more time on multimedia displays. On the other hand, a visitor classified as a fish will view a lower number of artifacts because they mostly stay at the center of the room and might not be interested in the recommenders either. Finally, the visitors characterized as a grasshopper are focused on some specific exhibits. Although they spend a considerable amount of time on the items of interest, they may end up viewing fewer artifacts on the whole. The ability to classify visiting behavior can help in the development of models which can adapt to different kinds of tastes and still offer the best output.

#### 3.1.2. The Popularity of Displayed Artifacts

While analytics of gathered IoT data has been helpful in classifying viewing behavior of visitors, these classifications cannot predict whether a certain exhibit will be a popular attraction or not.

The reputation of an exhibit computed by Cuomo et al. [24], is formulated as:

(1) $$rep_{E}\left(s\right)\approx \underset{1\leq V\leq N}{\sum }(bw_{V}\cdot perc_{V,E}\left(s\right))$$

where *rep_E_* is the reputation of the Eth exhibit at step *s* of the path taken by the visitor *V*, and *bw_V_* is the weight assigned to each category of the visitor. The weight values are assumed as *bw_V_* = 0 for the visiting style of a fish, *bw_V_* = 0.5 for a butterfly and grasshopper visiting styles, and *bw_V_* = 1 for the visiting behavior of an ant. The factor *perc_V,E_* is the percentage of artworks which visitor *V* has viewed by the time they reached the exhibit *E*. Although it can be seen that the visiting behavior has a direct impact on the reputation of a specific display exhibit, it is not the sole parameter. The path taken by the visitor and the placement of the exhibit also play an important role, as expressed by the parameter *perc_V,E_*.

Research has been carried out to determine the relationship between the duration of stay of visitors and the amount of footfall that an artwork witnesses [24]. The study assumed a short stay to be less than or equal to 60 min in duration, and the longest stay to be of a duration greater than 8 h. It was discovered that regarding the sequence of visiting the artifacts and the number of exhibits visited, the figures were comparable between the short stay and longest stay visitors. It was also observed that both of these groups preferred some specific paths to explore the museum, which might be attributed to the presence of very famous exhibits, favorable locations, or availability of escalators, or ease of access, for example. This observation might also be attributed to the presence of other visitors at an exhibit, crediting to the fact that humans follow others’ examples. Although we are aware of basic visiting patterns, it is required to observe the footfall which the artifacts receive to predict their popularity in comparison with other artifacts. The ability to predict the reputation of an artifact can give confidence to the museums to bid for related items and build on a theme around the popular items. The knowledge can also help the planners with identifying the role of artifact placement with respect to their reputation. This in turn can help in a better spatial organization so as to reduce crowding, and on the other hand, decrease the probability of an artifact being neglected due to poor placement. Keeping in mind the importance of the reputation of the display exhibits at a heritage site, the proposed framework aims to determine the viewers of an artifact and compare the density of viewers of an artifact against other artifacts through the clustering method [25].

### 3.2. Cluster Analysis

Cluster analysis of datasets, commonly referred as clustering, is a method to analyze a set of data points by grouping them over certain attributes [26,27,28,29]. Clustering forms the basis of unsupervised machine learning models because it can be applied to datasets in which attributes have not previously been labeled. Therefore, clustering can form the basis of building the attribute sets for an unknown dataset, because it can be used to reveal attributes based on the groupings of the data points. Based on the type of desired output and the method of forming the clusters of data points, clustering algorithms can be categorized into five major types. The first is based on partitioning, and the most popular clustering algorithm of this category is K-means clustering. The second is centroid-based partitioning which builds clusters over center points of each data group. One of the commonly used centroid-based algorithms is mean-shift clustering. The third type of clustering algorithm is model-based. Gaussian mixture model-based algorithms are the popular clustering algorithms of this type. To determine the parameters such as mean and standard deviation, expectation–maximization optimizations can be applied to the datasets. The fourth type of algorithm is hierarchical clustering. This algorithm works best when it is required to uncover a hierarchical relationship within the provided data points. The fifth kind of clustering algorithm is the density-based algorithm. This algorithm is well suited for cases where outliers are required to be included in the output. Due to the use of a dynamic and fast-changing dataset, a density-based clustering algorithm was used for the framework. Furthermore, the expected output aimed for a density-wise plot of footfalls around display exhibits. On the implementation front, the Plotly library [30] for Python was used to execute clustering over the X, Y coordinates of the detected human figures.

### 3.3. Human Figure Detection in Video Streams

In the proposed framework, detection of human figures has been utilized to study the visitor density around display exhibits at a heritage site, or a museum. Although several algorithms have been developed to distinguish a human figure, two major approaches come to the fore. The first is the spatio-temporal technique, which works on the distinct movements of a human body, also called the human gait [31]. This approach can be applied to video streams by dividing the streams into time slices and then recovering the parameters which characterize the human gait. The second approach is appearance-based algorithms, which detect silhouettes of human figures [32]. While this seems like a simpler approach, it is riddled with the possibility that the presented streams can have humans in multiple types of viewing angles, namely the top, or the side view, or the partial view. Video streams were sampled at periodic intervals; therefore, the more popular appearance-based approach was adopted for the implementation of ARTYCUL. Histograms of oriented gradients (HOG) have been one of the widely accepted object detection models [33,34]. Due to the wide support of this library, we have used HOG-based human detection Application Programming Interfaces (APIs) in the prototype development.

The human figure is a much more complex subject than the human face, and many well-tested algorithms have thus been developed. When we consider a human detection requirement in a real-time system, the method needs to be agnostic to the viewing angle of the camera. It should also be efficient enough to handle real-time feature descriptions. Finally, a good feature descriptor should be least affected by constraints of location, the brightness while recording, obstructions in the premises, etc. [35]. Research has been carried out to compare the performance of frame differencing-based algorithms, Circular Hough Transform-based and Histogram of Oriented Gradients (HOG)-based human detection methods. It was found that HOG-based counting of persons produced the best results with respect to obstructions and camera placements. In the cases where a HOG-based model implemented through a support-vector machine (SVM) was not trained for certain camera angles, the model was still found to yield the most accurate results [35]. The HOG feature descriptor breaks the image into cells and therefore is immune to geometric transformations and primarily works on the orientation of the review object.

Among the various descriptor techniques being used for human detection, the HOG feature descriptor has been found to result in the best performance [36]. The HOG feature descriptor operates in four major steps. Firstly, the three primary color channels—red, green and blue—are normalized through square root operation in addition with logarithmic compression. Therefore,

(2) $$Channel_{R}=\;\surd Channel_{R}$$

(3) $$Channel_{G}=\surd Channel_{G}$$

(4) $$Channel_{B}=\surd Channel_{B}$$

The next major step computes the gradients of the color intensity and their orientation, so as to elucidate the texture information while preserving the variations in brightness levels. The mask vector to calculate the gradient is given by mask = [−1, 0, 1], and its transpose vector for each of the color channel Channel_X_, where *X* = *R*, *G*, *B*.

The intensity of the resulting gradient will be,

(5) $$Intensity=Gradient_{X}^{2}+Gradient_{Y}^{2}$$

where *Gradient_X_* is the result gradient after applying mask [−1, 0, 1] and *Gradient_Y_* is the result gradient after applying mask [−1, 0, 1]^T^. The third major step results in the construction of histograms by binning together the gradient values computed in the previous step. The final step carries out normalization operation again, but this time over the histograms. This step results in blocks which are formed through this normalization of histograms.

By delving into the process of HOG-based feature extraction, we see that the method focuses on the contours and edges of the figures. This is a significant contributing factor to the robustness and accuracy of this model.

### 3.4. The Proposed ARTYCUL System

**Figure 1:**
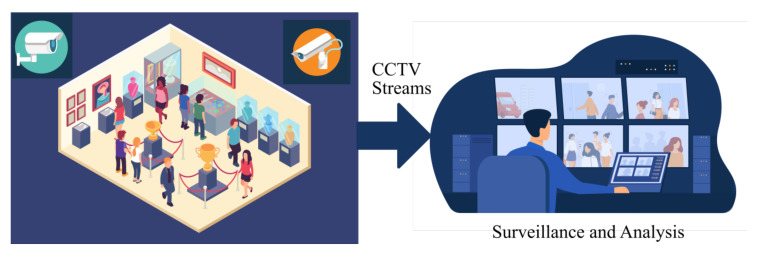
The proposed ARTifact popularitY for CULtural heritage (ARTYCUL) system and its position in a CCTV monitored premise.

This section elaborates on the conceptual architecture and implementation details of the prototype of ARTYCUL (ARTifact popularitY for CULtural heritage). The proposed system is based on applications of video streams received from closed-circuit televisions installed in a premise. As seen in Figure 1, the streams from the cameras are generally used for surveillance purposes at a centralized location. An instance of ARTYCUL could therefore be deployed at the monitoring station to further analyze the received visual information.

#### 3.4.1. ARTYCUL Architecture

ARTYCUL was conceptualized as a medium to visually provide popularity measures of a display item to the curators. It was designed to be easily navigable and readable.

**Figure 2:**
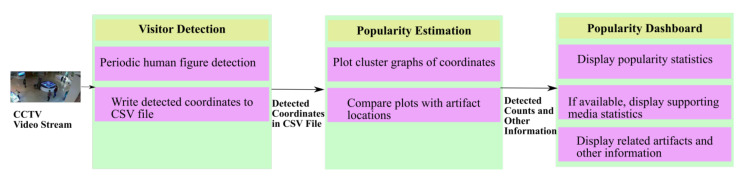
The architecture components and expected inputs and outputs between the modules of the proposed framework.

The architecture of the proposed framework can be divided into three modules, namely, the visitor detection, popularity estimation, and the popularity dashboard. Further components of the three modules are shown in Figure 2. The live video streams from the CCTV cameras are inputted to the visitor detection module to sample the stream at user-defined intervals. The idea behind the periodic detection was to take a snapshot of the visitors around the display exhibits at regular intervals. The use of the word snapshot is in the literal sense; the framework captured a video frame after every N min, where N was a configurable integer value. On this captured frame, human figures were detected and a list of coordinates for the detected human figures was saved to a CSV file. Therefore, it could be said that a snapshot of visitors was taken after every N minutes. It should be noted that the framework does not try to detect facial features or gender, but it simply works on detection of the complete or part of a human figure.

General operating hours of a museum or a cultural heritage site mostly total around 10–12 h. By setting N to an optimal integer value, an optimal dataset can be generated that is fit for use from day one. For instance, if N = 5, the video streams will be sampled every 5 min. A single frame sample can give multiple pairs of coordinates for the detected human figures. This results in 12 sets of human figure detections per hour. The detected coordinates are appended to a file for future use. If the personnel at the site wish to refresh the collected data, they can simply back up the CSV format file and let the framework create a fresh file to save new datasets.

The coordinates of the detected human figures were read from the CSV file by the popularity estimation module. This module used clustering of the X, Y coordinates from the file. The resulting graphical representation was an easy-to-read summary of visit patterns around the displayed artifacts. The clustered output can be matched against the fixed frame of a CCTV video stream. The ability to discern the different densities of persons visiting the artifacts does not require any advanced technological expertise, and can easily be used by museum curators and planners.

The third module, named popularity dashboard, was conceptualized to be a composite view of popularity details of the concerned item. It could offer the curators the option to select a specific artifact and see its relevant visualizations, such as visitor densities over the different hours of a day, or a table that summarized artifact details and links to related artifacts at the site. The relationship between artifacts could further uncover the placement strategies and content management of any available audio-visual aids.

#### 3.4.2. ARTYCUL Prototype

The prototype for ARTYCUL was implemented in Python language. The visitor detection module used the well-supported open-source library OpenCV [37], version cv2. The APIs of class HOGDescriptor were used to detect human figures in the video streams of security cameras and determine the X, Y coordinates of the detections.

Currently, two mainstream approaches are available for human figure determination and counting. The first approach is to detect individual objects, check the detected objects against classification labels, and, if they qualify, then count them. This method is the underlying principle of the HOG (Histogram of Oriented Gradients) feature descriptor. The other commonly used method to count objects is through background subtraction [38]. It works on the principle of assuming that the background is static and thus the comparison of two frames can be used to identify differences. In the case of videos which have been recorded in low light, an extra image noise needs to be removed for a better recognition [39]. ARTYCUL aimed at processing CCTV video streams; therefore, background subtraction would require extra computation and may not have given accurate results.

The efficiency of HOG descriptors has been the focus of a number of research. A HOG descriptor can be applied to both images and video frames. When we consider human figure detection, the images or video frames may contain human objects in three kinds of views. These are the top view, the side view, and the profile view. It has been found that the results for side views are least accurate. To achieve maximum coverage, security cameras are generally mounted at a height, and either at the center of the room or on one of the walls or in a corner. All such placements yield video frames which are optimal for usage by a HOGdescriptor. Another factor which works in the favor of HOG descriptors is that it provides an API detectMultiscale() [40], which offers multiple parameters for instance scaling, padding, and so on, to modify and thus improve the accuracy of the model.

The Python program for pseudo-code, summarized in Algorithm 1, was run against multiple video streams, of which we have listed three in the Section 4. There are various methods available that evaluate the performance of a machine learning model. We computed confusion matrices to check the accuracy of the framework. For this, a video frame was divided into cells for the easier examination of human presence or absence. The measure of performance is whether the model was able to predict human presence in a certain cell of the video frame correctly. The cell will be marked as 1 if a presence was detected; otherwise, it will be marked as a 0. The number of correct detections can be represented as *TRUE_P_* and detections found to be incorrect can be labeled as *FALSE_P_*. While the correct detection of absence of human figures can be labeled as *TRUE_A_*, the incorrect absence identified by the model has been labeled *FALSE_A_*. These individual values have been computed to populate the confusion matrix. The accuracy of the model can be computed as:

(6) $$Accuracy=\frac{\left(True_{P}+True_{A}\right)}{\left(True_{P}+False_{P}\right)+\left(True_{A}+False_{A}\right)}$$

**Algorithm 1** Pseudo-Code for visitor detection from a video streaming source1: Read CCTV video stream file 2: Set frame as video frame at time T, where T is the last epoch 3: Set scale as 1.05 for optimum human figure detection 4: Set group threshold as 1to detect each individual in frame 5: Detect and draw detection rectangles over identified human figures 6: **for** rectangle in detection rectangles of a frame 7:   Set X as x coordinate of top, left vertex of the rectangle 8:   Set Y as y coordinate top, left vertex of the rectangle 9:   Set width as width of the rectangle 10: Set height as height of the rectangle recommendations 11: Set center_x as X + width/2 12: Set center_y as Y + height/2 13: Save center_x, center_yin CSV files //the CSV files will be read by Popularity estimation module 14: **end for**

## 4. Results

The Python implementation of the ARTYCUL prototype was tested on several video feeds provided by IP security cameras. Each of these had a different viewing angle and varying levels of footfall with respect to the camera frame. For the experiments, the size of video frames was 1080 × 1920, in accordance with the resolution of the cameras used for the surveillance of premises. A video frame was divided into cells, namely A to F for rows and numbers 0 to 7 for columns as shown in Equations (7) and (8).

(7) $$Height_{row}=\frac{1080}{6}pixels=180\;pixels$$

(8) $$Width_{column}=\frac{1920}{8}pixels=240\;pixels$$

Therefore, a specific point in a video frame can be addressed using the row values lying between A to F, and column values lying from0 to 7. To define a finer level of granularity, the frame could further be divided into larger number of rows and columns.

**Figure 3:**
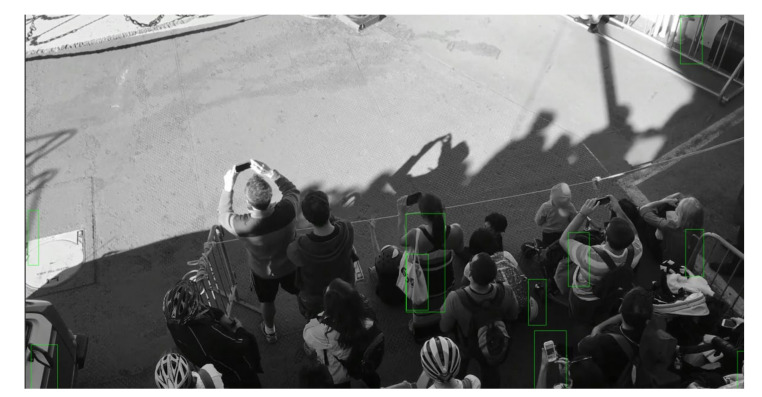
Screenshot of video stream being analyzed by the Python script and its division into labeled cells.

For the first experiment, we used a video stream where the footfalls happened at one end of the *Y*-axis of the video frames. The screenshots in Figure 3 show that the crowd was limited to one side of the field of vision. As seen in the figure, the green rectangles were the created by the HOG descriptor for detected human figures. The machine learning model underlying the HOG descriptor is based on an SVM; therefore, it took a couple of iterations to achieve more accurate results, while it quickly built on the dynamic dataset.

**Figure 4:**
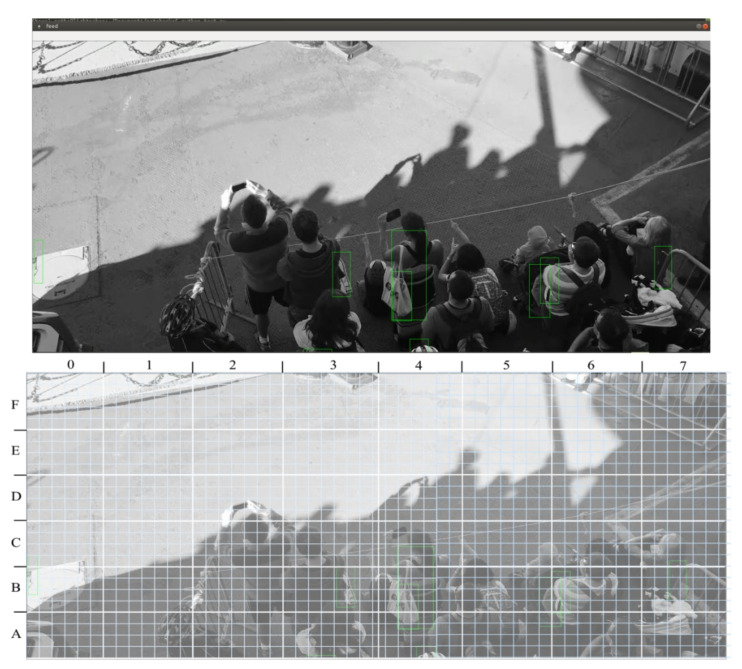
Another video frame of the test stream labeled into cells.

A frame for the same stream with the detailed cell division is shown in Figure 4.

If we again specify the cells which should give minimal human detections, the rows labeled *E* and *F* should give minimum possible human detections for all columns. To put this assumption in the form of an equation:

(9) $$P\left(r,c\right)\approx 0,\;where\;E\leq r\leq F\;and\;0\leq c\leq 7$$

The density of persons in the frames was approximated through clustering. The scatter plot of the saved detection coordinates to show the clustering is shown in Figure 4. The graph is easy to discern by a person with limited technical knowledge.

**Figure 5:**
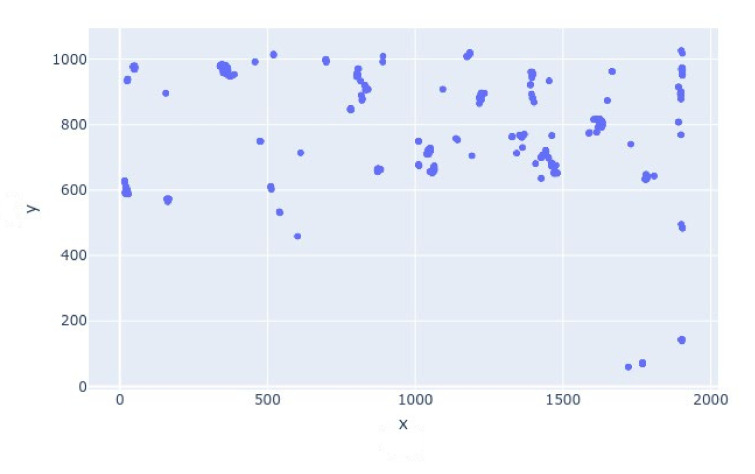
The clustered graphical representation of the detections done by the proposed framework.

**Figure 6:**
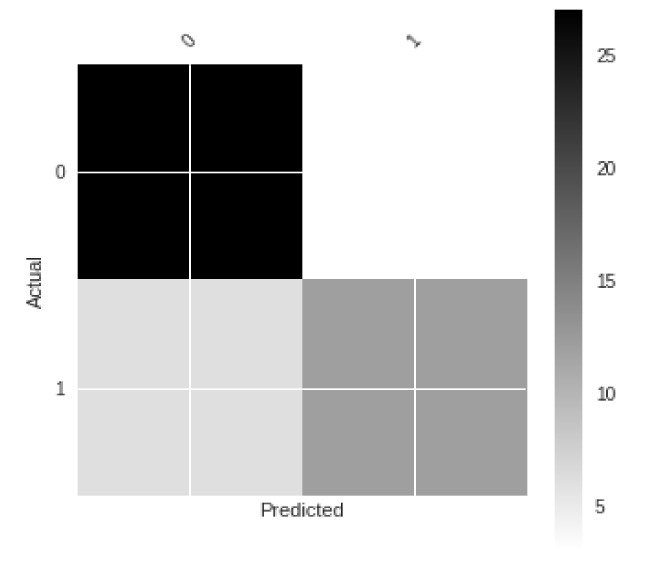
The plotted confusion matrix for detections done by the framework.

Using Figure 5 and Figure 6, a confusion matrix was plotted to discern accuracy of detections of human presence in the video frames. The resulting confusion matrix in numerical form was computed and is shown in Table 2.

The confusion matrix for Table 2 is shown in Figure 5.

Next, we considered the remaining cells after excluding the perimeter, which were again found to be 24 in number. The following labeled cells were checked for result accuracy: B(1–6), C(1–6), D(1–6), and E(1–6).The resulting confusion matrix in numerical form is shown in Table 3.

**Figure 7:**
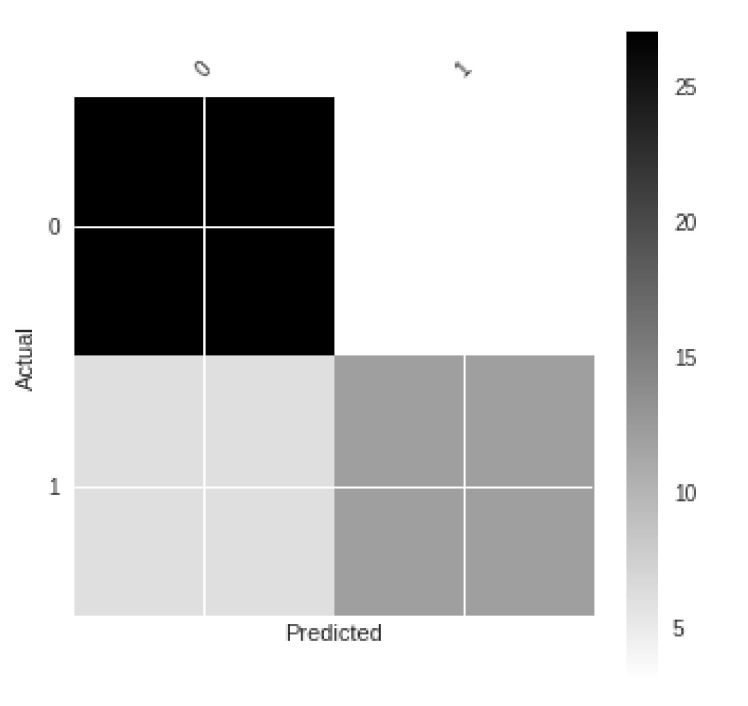
Confusion matrix for detections done on one-sided dense video frames.

The confusion matrix was plotted using Matplotlib and is shown in Figure 7.

**Figure 8:**
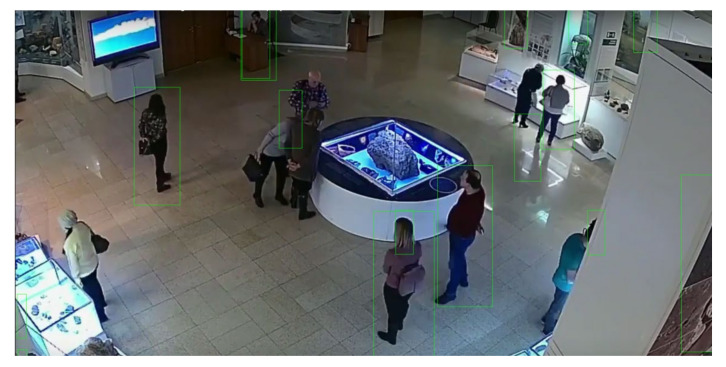
The detection logic output for a video stream of a Russian museum.

Another freely available video stream of a Russian museum was tested using the ARTYCUL prototype. The detections are shown in Figure 8.

**Figure 9:**
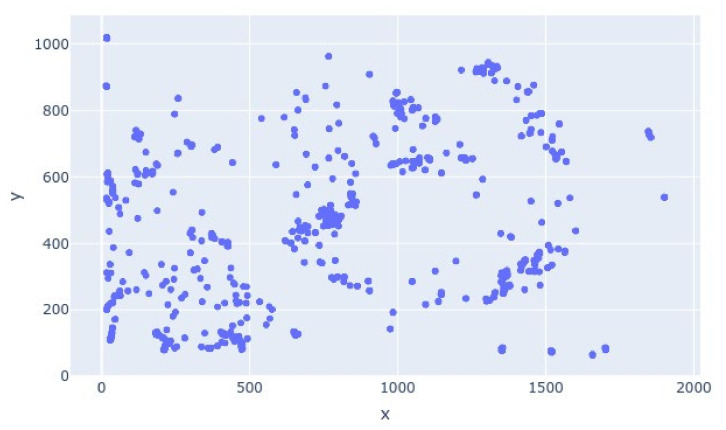
The scatter plot of the coordinates of detected human figures.

The scatter plot of the detected figures is shown in Figure 9.

**Figure 10:**
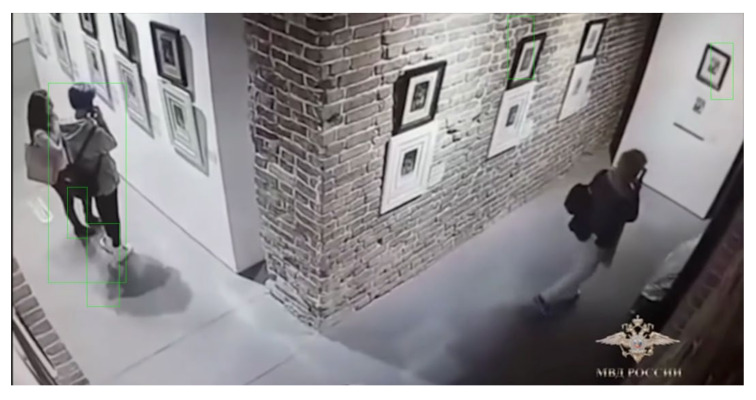
The detection logic output for a video stream.

**Figure 11:**
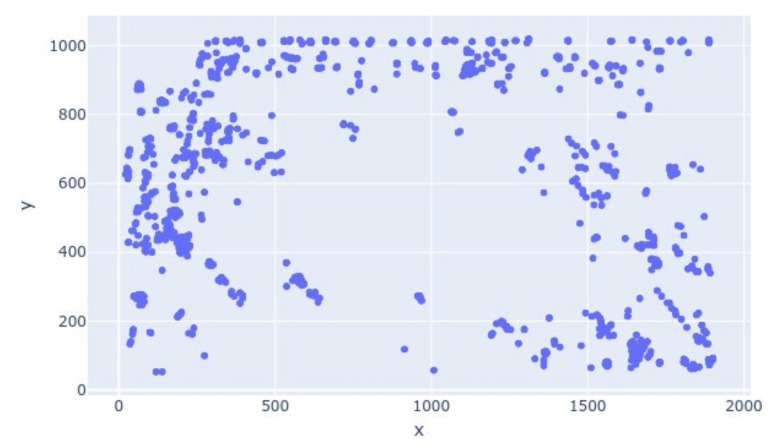
The detection logic output for a video stream.

In Figure 9, the middle of the plotted graph that corresponds to an artifact in the video stream is seen without any human presence, while around the artifact, dense human presence can be observed from the clustered points. Another video stream from the International Arts Centre in Yekaterinburg was seen to be demarcated by a wall, and visitors had to walk on a certain part of the field of vision. Figure 10 shows a screen grab of the stream with the detection rectangles drawn by Python script. The clustered output in Figure 11 shows no human detection in the coordinates that mapped to the division wall.

**Figure 12:**
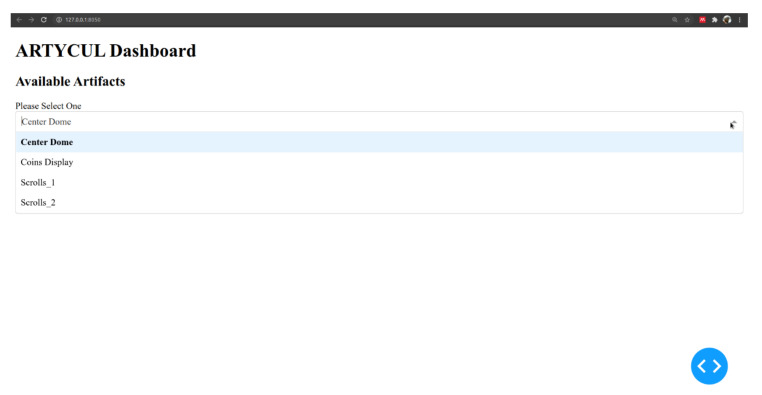
A prototype of the popularity dashboard module of ARTYCUL.

**Figure 13:**
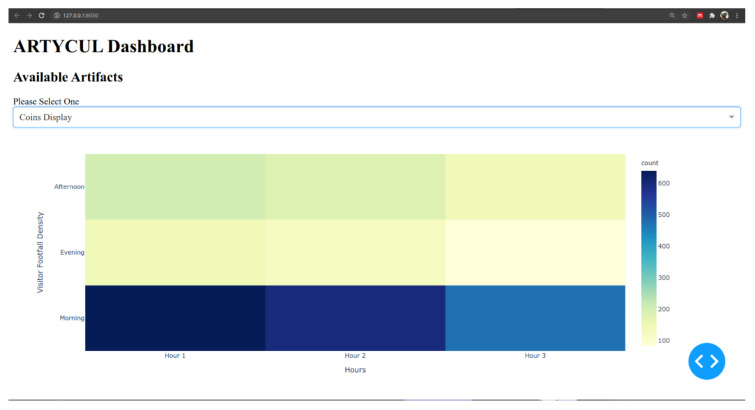
A prototype of the popularity dashboard module of ARTYCUL.

The third module of ARTYCUL was designed to provide the curators with an easy-to-use dashboard to view artifact-related numbers. Figure 12 shows the prototype of the dashboard developed using Dash components to display visitor densities over the operational hours. For instance, a functional day at a museum could be assumed to be from 09:00 AM until 18:00 PM. The operational hours could be divided into morning, afternoon and evening with each spanning over three hours, and then the visitor densities can be shown as in Figure 13.

For the example shown in Figure 12, the sample artifact boasted maximum visitors during the morning hours, while evening hours saw fewer people.

To further indicate the significance of ARTYCUL in artifact curation, we used the Melbourne Museum dataset generated and used in a previous study [41] that investigated multiple aspects of artifact placement and visitor behavior. The dataset detailed time-annotated visits of 158 unaccompanied males who had visited the museum. The total visit durations spanned over approximately 291 h, out of which the viewing time was estimated to be 240 h. The exhibit areas of Melbourne Museum included diverse topics such as Aborigine heritage, food technology, marine life, flora fauna typical to Australia, evolution, and so on. The research by Bohnert and Zukerman was relevant to ARTYCUL, because it suggested that a visitor’s prospective viewing duration was in direct correlation with semantic distances of the exhibits, rather than the exhibits that had been already viewed by the visitors. Thus, spatial factors such as placement form an important role in the viewing predictions.

The acquired dataset contained exhibit area names and viewing duration in seconds for anonymized study participants. It was found that the majority of exhibit areas were related. For instance, the exhibit area called “ABugsLife” could also be related to exhibit areas named “BugsInTheMuseum” and “WorldOfBeetles”. Table 4 shows a small subset of the relationship matrix between different exhibit areas.

**Figure 14:**
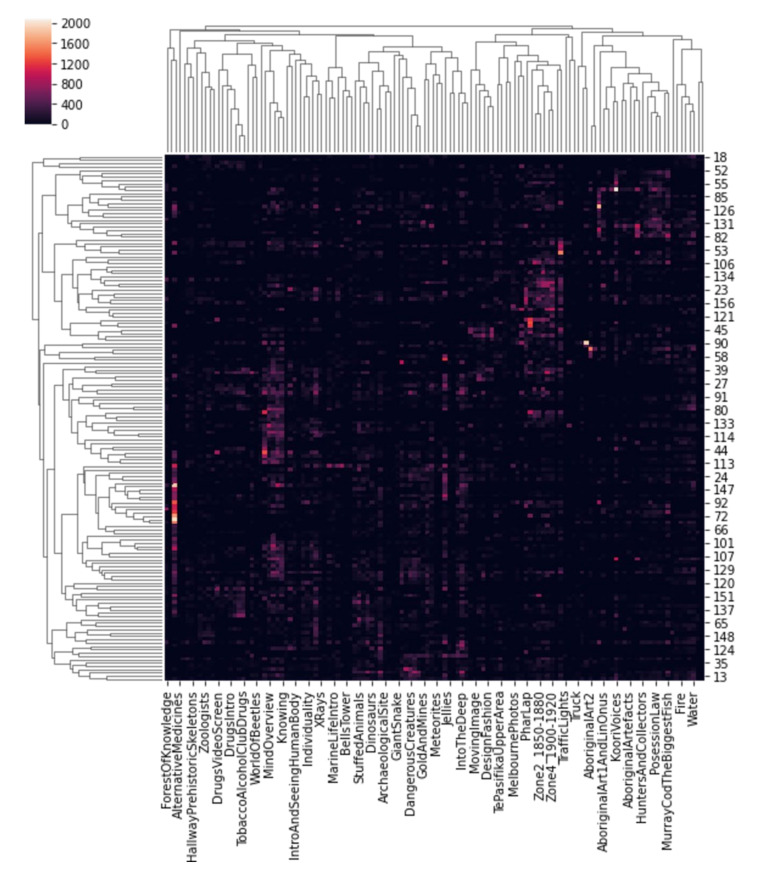
Clustering of Melbourne Museum exhibit areas based on correlations at different levels of hierarchies and visit durations.

To further display the correlation-based clustering of the exhibit areas, a hierarchical dendrogram for the correlation was plotted. In Figure 14, it can be seen that many exhibits show correlation in terms of the visit durations and themes.

**Figure 15:**
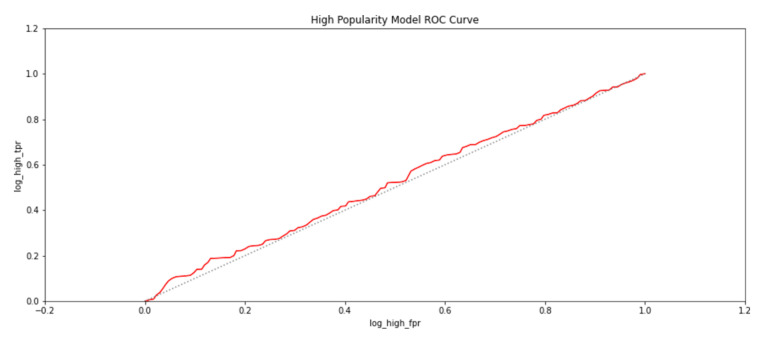
The probability of an exhibit area being in a highly popular category as identified by a logistic regression-based classifier.

**Figure 16:**
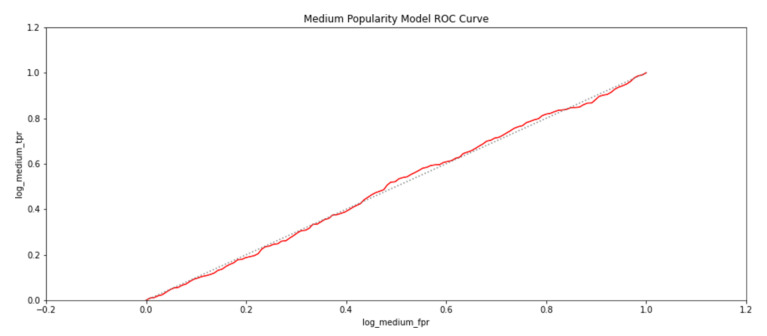
The probability of an exhibit area being in a medium popular category as identified by a logistic regression-based classifier.

**Figure 17:**
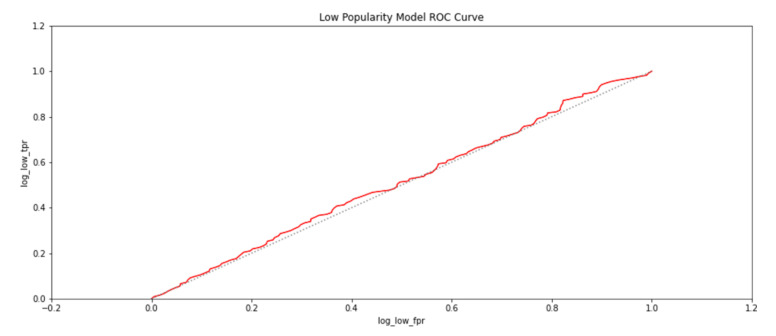
The probability of an exhibit area being in a less popular category as identified by a logistic regression-based classifier.

To further establish the relationship between spatial properties of the exhibits and their popularity, logistic regression was carried out to determine if the presence of an exhibit could be predicted for a given popularity class. The popularity classes were categorized as highly popular exhibit areas if a participant had viewed it for more than a minute. An exhibit would assumed to be moderately popular if it had been viewed for more than thirty seconds but less than a minute. Otherwise, the exhibit would be deemed to have a low popularity, because it had been viewed for less than thirty seconds. The trained regression models for the three popularity classes gave an accuracy of 71.8%. The metrics of true positive rates and false positive rates of the modeled regression-based classifiers were also used to plot receiver operating characteristics (ROC) and are shown in Figure 15, Figure 16 and Figure 17. The grey line in the ROC plots distinguishes the 50% probability of class identification. Therefore, a logistic regression algorithm could correlate an exhibit area with the popularity category with which it was associated for more than 50% of cases.

The analysis of the Melbourne Museum dataset pointed to the significance of spatial parameters as an impacting factor towards the visit potential of an artifact. The visit densities detected by ARTYCUL can be used to discern the popularity class of an exhibit area and whether it qualifies as highly popular, moderately popular, or least popular. A scarce density of visitors around an artifact would translate to visitor disinterest, which could lead curators to re-consider its placement in the premise or to remove it altogether. Another interesting application of ARTYCUL could drive recommender systems that were not dependent on user profiling, thus alleviating fears of unsolicited communications or information breaches.

## 5. Discussion

Cultural heritage is an identity of a nation and requires continuous efforts for its preservation and dissemination among the masses. Therefore, it is an important domain for the application of technology, and IoT has emerged as one of the most promising mediums. The advances in IoT have been incorporated into the CH domain for some time now. The developed systems were aimed at enriching the experience for the visitor. The proposed framework, on the other hand, was conceptualized as a non-invasive, backward-compatible system to discern the popularity of displayed artifacts and therefore facilitate a better planning of the site.

The results produced in Section 4 show that an algorithm for human figure detection and classic machine learning algorithm of clustering can be combined to implement a system to provide an easily discernable representation of artifact popularity. This, in turn, can help the curators to improve on-site organization and thereby enhance the prospects of any neglected items. The proposed framework does not require any extra hardware or installation costs; only the video streams of the pre-installed CCTV cameras. The system can be further extended to drive a recommender system to direct visitors towards related items at the same site, or to any other partner premise.

The prototype of ARTYCUL was implemented using OpenCV library which uses a support vector machine (SVM) as the underlying classifier for human figure detection. The use of an SVM classifier provides the best possible accuracy among classification algorithms, but it is riddled with the initial delay due to the gradual training examples which an SVM classifier uses to build its performance. Additionally, the authors tested the prototype for ARTYCUL using OpenCV due to its availability as an open-source library, well-supported documentation, and low computational requirements. It should be noted that more powerful human-figure tracking solutions are available and could be used for development of the proposed framework.

## 6. Conclusions

This report is an endeavor to understand the utilization of IoT-based technology in the CH domain. Several sophisticated systems installed at heritage sites have ensured a fulfilling and multimedia-driven visit experience. These smart systems are driven by IoT sensors and local networks to take measurements, track visitor behavior and communicate with their personal devices or those installed in the premises. The visitors acquire more information through recommenders and other audio-visual aids. To drive such smart systems, it is also required to be able to predict the reputation of a display artifact. The proposed framework has been designed to compare the popularity of display artifacts in the simplest of forms, so the results can be discerned without any technological knowledge.

ARTYCUL is a simple framework that gives a cluster-based graphical representation to the popularity of display artifacts at a cultural heritage. While the framework utilized an SVM to determine the human figures and density-based clustering to create an unsupervised representation of visiting patterns, it could be further developed to include the classifications of visitor behavior to obtain more accurate results. It should be noted that the HOG descriptor feature detection mechanism does not work well on blurry videos. Additionally, this framework will require a proper maintenance of the cameras that provide the video streams to the system.

On a positive note, this framework can be applied to the existing network of CCTV cameras installed in premises. Therefore, the cost of additional hardware is virtually zero. The framework does not require dedicated internet bandwidth. It also does not require any visitor information or building of profiles; as a result, there is essentially no possibility of compromises on visitor data privacy.

## Figures and Tables

**Table 1:** Existing applications of IoT in cultural heritage domain.

Real-World Application	Example	Challenges Faced
Monitoring and control of smart network in the premises	Smart system at Maschio Angioino castle in Naples for ambient lighting	Should not overwhelm the non-technical users
Provide an interactive visit experience	Smart system accesses handheld devices, either owned by the site or the visitors, to track visitor interest and publish relevant information	User privacy concerns and maintenance requirements of the sophisticated hardware
Analytics of data generated by an IoT-based smart environment	Visitor behavior is classified and used to determine the popularity of the cultural artifacts on display	Development of sophisticated models requires technical expertise

**Table 2:** Confusion matrix for detections done by the framework.

True Condition			
**Predicted** **Condition**		True (Presence)	False (Presence)
	True (Actual)	0.9	0.16667
	False (Actual)	0.2	0.66667

**Table 3:** Confusion matrix for detections done in the inner cells of video frame.

True Condition			
**Predicted** **Condition**		True (Presence)	False (Presence)
	True (Actual)	0.9	0.16667
	False (Actual)	0.2	0.66667

**Table 4:** Subset of ascertained exhibit area relationships for use in the analysis.

	1930sStandingUpForRights	ABugsLife	AboriginalArt1AndLinOnus	AboriginalArt2	AdvertisementADayInPompeii	Aliens
1930sStandingUpForRights	Y	N	N	N	N	N
ABugsLife	N	Y	N	N	N	N
AboriginalArt1AndLinOnus	N	N	Y	Y	N	N
AboriginalArt2	N	N	Y	Y	N	N
AdvertisementADayInPompeii	N	N	N	N	Y	N
Aliens	N	N	N	N	N	Y

## Data Availability

Data available in a publicly accessible repository.
